# An Update on the Role of Nrf2 in Respiratory Disease: Molecular Mechanisms and Therapeutic Approaches

**DOI:** 10.3390/ijms22168406

**Published:** 2021-08-05

**Authors:** Jooyeon Lee, Jimin Jang, Sung-Min Park, Se-Ran Yang

**Affiliations:** Department of Thoracic and Cardiovascular Surgery, Kangwon National University, Chuncheon 24341, Korea; 331joo@naver.com (J.L.); jangjim@naver.com (J.J.)

**Keywords:** Nrf2, keap1, oxidative stress, ROS, respiratory diseases, lung

## Abstract

Nuclear factor erythroid 2-related factor (Nrf2) is a transcriptional activator of the cell protection gene that binds to the antioxidant response element (ARE). Therefore, Nrf2 protects cells and tissues from oxidative stress. Normally, Kelch-like ECH-associated protein 1 (Keap1) inhibits the activation of Nrf2 by binding to Nrf2 and contributes to Nrf2 break down by ubiquitin proteasomes. In moderate oxidative stress, Keap1 is inhibited, allowing Nrf2 to be translocated to the nucleus, which acts as an antioxidant. However, under unusually severe oxidative stress, the Keap1-Nrf2 mechanism becomes disrupted and results in cell and tissue damage. Oxide-containing atmospheric environment generally contributes to the development of respiratory diseases, possibly leading to the failure of the Keap1-Nrf2 pathway. Until now, several studies have identified changes in Keap1-Nrf2 signaling in models of respiratory diseases, such as acute respiratory distress syndrome (ARDS)/acute lung injury (ALI), chronic obstructive pulmonary disease (COPD), idiopathic pulmonary fibrosis (IPF), and asthma. These studies have confirmed that several Nrf2 activators can alleviate symptoms of respiratory diseases. Thus, this review describes how the expression of Keap1-Nrf2 functions in different respiratory diseases and explains the protective effects of reversing this expression.

## 1. Introduction

Reactive oxygen species (ROSs) have free radicals, including superoxide anions and hydroxyl radicals, and active non-radical oxygen species, including hydrogen peroxide and nitroperoxide, which are generated by the partial reduction in oxygen [1]. In brief, ROSs are a set of unstable molecules. Recently, it has been reported that ROSs play a crucial physiological role in cellular homeostasis as well as various human diseases [2]. Moderate amounts of ROSs act in pathogen resistance and cellular signaling related to cell proliferation, metabolism, and differentiation [3], whereas high levels of ROSs induce pathology by damaging lipids, protein, and DNA [4]. In respiratory diseases, excessive ROSs are typically recognized to implicate the development of chronic, inflammatory, or aging-associated diseases: chronic obstructive pulmonary disease (COPD), idiopathic pulmonary fibrosis (IPF), acute lung injury/acute respiratory distress syndrome (ALI/ARDS), or lung cancer [5]. The balance of oxidants/antioxidants is maintained for the healthy status [6]; however, persistent or hyperinflammatory injuries can upset the balance of oxidative homeostasis in the tissue, resulting in the development of diseases [7]. In mammalian cells, it has developed cytoprotective mechanisms that abrogating ROS production via nuclear factor erythroid 2-related factor (Nrf2)/Kelch-like ECH-associated protein (Keap)1 pathway signaling.

## 2. Keap1-Nrf2 Signaling Pathway

**Figure 1:**
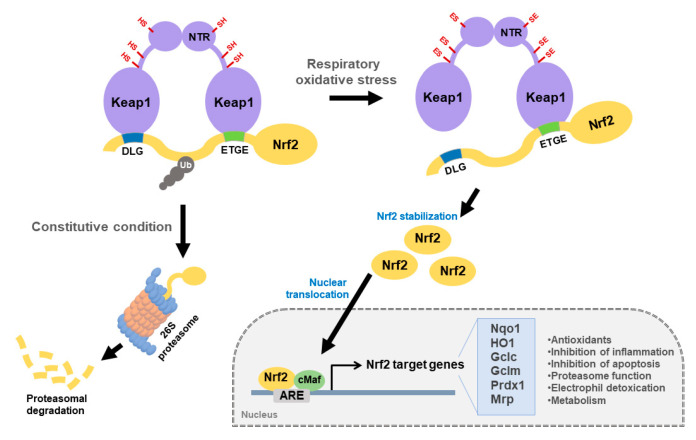
The Keap1-Nrf2 signaling pathway. Nrf2, a transcription factor, contributes to expression of cell-protective genes in response to respiratory oxidative stress. Keap1 is a regulatory protein that inhibits activation of Nrf2. Under Constitutional condition, Nrf2 remains bound to Keap1 and is degraded by the ubiquitin-proteasome pathway dependent on keap1. In respiratory oxidative stress environment, Keap1 is inactivated while Nrf2 is stabilized. The stabilized Nrf2 performs nuclear translocation and heterodimerizes with cMaf to activate target genes for cell protection through ARE. Nrf2 target genes include NAD(P)H quinone oxidoreductase 1 (Nqo1), heme oxygenase 1 (HO1), glutamate-cysteine ligase catalytic subunit (Gclc), glutamate-cysteine ligase modifier subunit (Gclm), Peroxiredoxin 1 (Prdx1), and Multidrug resistance-associated protein (Mrp) and perform antioxidants, inhibition of inflammation, inhibition of apoptosis, Proteasome function, electrophile detoxication, and metabolism.

Nuclear factor erythroid 2-related factor (Nrf2) is a transcription factor involved in oxidative stress response. Nrf2 plays an important role in the regulation of ARE-mediated expression of phase II detoxifying antioxidant genes, including heme oxygenase 1 (HO-1), superoxide dismutase 1 (SOD1), glutathione (GSH), UDP-glucuronosyltransferases (UGTs), sulfotransferases (SULTs), and N-acetyltransferases (NATs) [8,9,10]. Keap1 is a redox-regulated substrate adapter protein that controls cellular localization and the amount of Nrf2 within a cell [11]. Nrf2 consists of six domains, including the Keap1 binding domain (Neh2) and leucine zipper domain (Neh1). The N-terminal BTB domain of Keap1 binds to Cullin 3 (Cul3), Rbx1, whereas the C-terminal Kelch repeats domain binds to Nrf2 [12]. Normally, one Nrf2 can bind with a Keap1 homodimer or two Keap1 molecules by exploiting two biding sites, the DLG motif and ETGE motif, within the Neh2 domain. After binding with Keap1, Nrf2 is rapidly ubiquitinated and then proteasomal degraded by Keap1-Cul3 E3 ligase in the cytoplasm [13,14]. However, when exposed to electrophiles or ROSs, the cysteine residue of KEAP1 is deformed, the ubiquitin E3 ligase activity of the KEAP1-Cul3 complex is reduced, and Nrf2 is activated [15]. Activated Nrf2 is separated from Keap1 and translocates to the nucleus and is heterologous to the sMaf protein, and binds to ARE that is crucial for transcriptional activation of antioxidant genes, such as NAD(P)H quinone oxidoreductase 1 (NQO1), glutathione S-transferase (GST), and HO-1 [16]. Although Keap1-Nrf2 pathway signaling is strictly controlled as a protective response, its activity is frequently dysregulated in damaged tissues, leading to oxidative stress (Figure 1).

During respiration, humans inhale oxygen-containing air that enters the airways, passes through the bronchi, and eventually reaches the alveoli [17]. The inhaled oxygen travels into the bloodstream, whereas carbon dioxide travels into the alveoli via the gas exchange between the bloodstream and alveoli [18]. Lungs are enormously complex organs that constitute a hierarchical structure and consist of approximately 40 different resident cell types necessary to ensure respiration [19]. In the case of alveoli, these comprise two types of pneumocytes and phagocytic cells [20,21]. Type I epithelial cells (AEC1) maintain an alveolar structure where gas exchange occurs, whereas type II epithelial cells (AEC2) produce and secrete pulmonary surfactant proteins and can transform into AEC1 as a repair process against lung injury [22]. A network of capillaries surrounds the alveolar membrane and interacts with AEC1 for gas exchange to occur. Finally, alveolar macrophages move in the lumen of the alveoli, maintaining immune homeostasis [23]. Since oxidative stress and its downstream pathway might be independent responses to the same stimulation, the Keap1-Nrf2 pathway can be differently involved in every cell under oxidative stress. Given that the human respiratory system is structurally complex and directly exposed to exogenous sources of ROSs, such as cigarette smoke (CS), infectious organisms, or particulate matter (PM) in air, it is important to understand ROSs and the corresponding defense mechanisms in respiratory diseases. Therefore, this review discusses the role of the Keap1-Nrf2 signaling pathway, an antioxidative defense system, in various respiratory diseases.

## 3. Keap1-Nrf2 in Respiratory Diseases

### 3.1. ARDS/ALI

ARDS and ALI can result in respiratory failure and are conditions that cause inflammation in the lungs as well as dyspnea and cyanosis [24]. ARDS has several causes, including pneumonia, sepsis, trauma, and excessive blood transfusions, and has recently started being known as one of the clinical symptoms of COVID-19 [12,25]. ARDS activates an innate immune response pathway through pathogen-associated molecular patterns (PAMPs) receptors, such as toll-like receptor 4 (TLR4), which recognizes common pathogens and triggers an immune response. It leads to the secretion of inflammatory cytokines and chemokines and recruits lung macrophages and neutrophils into alveoli [26]. During this process, the permeability of the alveolar-capillary barrier is increased, causing pulmonary edema and alveolar epithelial damage. This leads to impaired lung compliance and gas exchange [27]. ARDS does not have a clearly defined therapy due to different causes and complex pathophysiology that have not been fully identified, resulting in a significantly high mortality rate.

Although the ARDS pathological mechanism is not fully identified, it has been steadily reported that ARDS is closely related to oxidative stress and inflammation. TLR4 signaling by PAMPs, such as lipopolysaccharide (LPS), regulates nuclear factor kappa-light-chain-enhancer of activated B cells (NF-κB) and NADPH oxidase 4 (NOX4), causing overproduction of ROSs and recruitment of inflammatory cells into the lungs and leading to lung damage [28,29]. Among them, several articles have recently demonstrated that Nrf2 is involved in oxidative stress and inflammation in ARDS. Several studies have shown that SNP of Nrf2 is associated with the severity of ARDS [30,31]. In recent studies that examined LPS-induced ARDS in murine models, activation of Nrf2 protected against ARDS by regulating macrophage polarization as well as oxidative stress [32]. In the hyperoxia-induced lung injury mouse model, Nrf2^−/−^ mice also showed higher macrophages and protein concentrations in bronchoalveolar lavage fluid (BALF) than in Nrf2^+/+^ mice. In addition, the activity of NQO1 required to inhibit the production of radical species was also dependent on Nrf2 [33]. Recently, activation of Nrf2 in ARDS patients with COVID-19 has been confirmed to have clinical benefits [34,35]. Activation of Nrf2 has been reported to reduce the severity of the cytokine storm in patients affected by COVID-19 [36,37]. Interestingly, activation of Nrf2 by using low-dose radiotherapy has previously been considered in various inflammatory diseases [35,38]. In addition, the interaction between Nrf2 and Keap1 is important to understand the oxidative stress defense mechanism in ARDS/ALI. Recently, ARDS induced by traumatic lung injury (TLI) has been associated with the Keap1-Nrf2-ARE signaling pathway [39]. In TLI-ARDS, the Keap1 level was increased, whereas Nrf2 and ARE levels were decreased. Furthermore, activation of Nrf2 improved arterial blood oxygenation and reduced the level of inflammatory cytokines. Additionally, various strategies have been tried to protect patients against ARDS/ALI by activating Nrf2. It has been reported that human placental mesenchymal stem cells of fetal origin (hfpMSC) protect alveolar epithelial cells through antioxidant effects. In the co-culture system of hfpMSC and A549, apoptosis was significantly reduced in A549 via accumulation of Nrf2 and reduction in Keap1. Therefore, hfpMSC can control the Keap1-Nrf2-ARE signaling pathway, resulting in inhibition of apoptosis and protection against oxidative stress damage in pulmonary epithelial cells [40]. Similarly, a recent study has reported that lung damage was inhibited in the LPS-induced ARDS model by bone marrow-derived mesenchymal stem cells. These cells have been shown to protect the lungs from oxidative stress through increased expression of Nrf2 and HO-1 [41]. Synthetic triterpenoid compounds CDDO-imidazole (CDDO-Im) activates Nrf2 by breaking the binding of Keap1-Nrf2. Treatment of CDDO-Im inhibited inflammatory response via increase in expression of Nrf2-dependent genes, such as (glutamate-cysteine ligase catalytic subunit) GCLC, NQO1 and (glutathione peroxidase 2) GPX2 in hyperoxia-induced lung injury mice [42]. Although Nrf2 deficiency impairs the alveolar repair mechanism and hyperoxia-induced DNA damage, GSH supplementation is effectively restored by inhibiting inflammation after hyperoxia [43]. Naphthalene-sulfonamide derivative SCN-16 has been reported to inhibit interaction with Keap1 and Nrf2, which reduced lung damage. SCN-16 increased nuclear Nrf2, HO-1, and NQO-1 and significantly reduced inflammation in the LPS-induced ALI model [44]. Moreover, treatment of sulforaphane after hyperoxia exposure not only inhibited lung injury but also improved energy metabolism in mitochondria and cardiovascular function in mice [45]. It has recently been confirmed that one of the tyrosine kinase inhibitors, dasatinib, has a therapeutic effect on ARDS. In the LPS-induced C57BL/6 model, administration of dasatinib inhibited the increase in neutrophils and macrophages triggered by LPS. It has also been shown to control the polarization of macrophages by increasing protein expression of Nrf2 and HO-1 [46]. Recently, it has been reported that oridonin increases expression of Nrf2, HO-1, and glutamate-cysteine ligase modifier subunit (GCLM) mediated by Akt and MAPK in RAW 264.7 cells. Interestingly, it was confirmed that the effects of oridonin remained the same in RAW 264.7 cells, which were induced to LPS after treatment of Nrf2 inhibitor brusatol. This suggests that oridonin not only has an antioxidant effect by increasing expression of Nrf2 via MAPK and Akt pathway but also has an anti-inflammatory effect by inhibiting NF-κB pathways independently of Nrf2 [47]. Several plant extracts, such as isorhapontigenin and TADIOS, have also been reported to activate Nrf2 in ARDS models [48,49]. In addition, small molecular compounds, such as cordycepin and syringin, caused Nrf2 activation in ARDS animal models [50,51,52].

### 3.2. COPD

COPD is a respiratory disease currently listed as the fourth leading cause of death. Its major manifestations include gradual airflow restriction, emphysema, and chronic bronchitis [53]. The pathological mechanism of COPD leads to inflammation and lung tissue destruction due to various processes, including the influence of genetic and environmental factors [12]. In particular, smoking is one of the main causes of COPD since cigarettes contain many oxidizing agents and various harmful substances that cause oxidative stress [54].

Direct or indirect CS exposure can cause oxidative stress and lung inflammation. The expression of Nrf2 protects against oxidative stress caused by excessive exposure to oxidizers in CS. High expressions of Nrf2 and related genes, such as HO-1 and GCLC, have been identified in blood mononuclear cells of COPD patients. In addition, patients with low forced expiratory volume in 1 s (FEV1) generally have higher Nrf2 and HO-1 expressions than patients with higher FEV1 [55]. Among COPD patients, those who smoke still have higher levels of Nrf2 target genes in bronchial epithelial cells than those who quit smoking [56]. A recent study has confirmed that in the porcine pancreatic elastase (PPE)-induced COPD model, Nrf2 is active due to translocation of Nrf2 from the cytoplasm to nucleus, and Keap1 expression is reduced [57]. In the PPE-induced COPD model, Nrf2^−/−^ mice showed higher COPD sensitivity than wild-type (WT) mice [58]. In addition, Nrf2 activators reduced emphysema, apoptosis signaling, oxidative stress, and pulmonary hypertension in WT models than in Nrf2^−/−^ models [59]. Transplanting Nrf2-positive bone marrow cells in Nrf2^−/−^ PPE-induced COPD model has been shown to weaken lung type and inflammation [60]. Administration of CS extract (CSE) in lung epithelial cells also activates damage-associated molecular pattern (DAMP) signaling with NF-κB, JNK1/2, ERK1/2, and p38 MAPK pathways, which involve translocation of Nrf2 into the nucleus [57,61]. Interestingly, the expression of Nrf2 is associated with receiver for advanced glycation end products (RAGE) signaling. RAGE is a multiligand transmembrane receptor that acts as a pattern recognition receptor [62]. RAGE has a variety of isoforms through alternative splicing, of which the most dominant expression of full-length membrane-bound RAGE is high in lung tissue of patients with COPD and PPE-induced COPD mouse models. Treatment with a RAGE antagonist in a PPE-induced COPD mouse model inhibited the expression and DAMP signaling of RAGE. On the other hand, treatment with RAGE antagonists in the PPE-induced COPD mouse model did not relieve emphysema symptoms in Nrf2^−/−^ mice [57]. The absence of Wnt prevents the accumulation of β-catenin by a destructive complex consisting of GSK, Axin, and APC. Conversely, activation of Wnt causes activation of TCF/LEF transcription factors due to the accumulation of β-catenin [52]. Recently, Wnt/β-catenin activation has been reported to be reduced in most patients with COPD [63]. In recent studies, the treatment with Wnt activators in the PPE-induced mouse models reduced the severity of emphysema and IL6 level, but not in Nrf2^−/−^ mice. Nrf2 expression was also higher in the Wnt activators treatment group than in the non-treatment group [64]. This suggests that activation of the Wnt signaling pathway protects against pulmonary inflammation and emphysema by activating Nrf2.

These studies show that research is continuously underway to improve the treatment of COPD by activating Nrf2. Sulforaphane (SFN), a *Brassica oleracea* extract, has been previously reported to inhibit oxidative stress by promoting activation of Nrf2 in various disease models, including COPD [65,66,67]. However, for 89 patients with COPD, the antioxidant effect of SFN was not significant. The dose of SFN did not significantly change mRNA levels of Nrf2, HO-1, Keap1, and NOQ-1 in alveolar macrophages and bronchial epithelial cells. In addition, the amount of cytokines in serum and bronchial alveolar lavage (BAL) has not changed substantially [68]. It has recently been reported that triterpene acids (TAs) can have a beneficial effect on cigarette-induced COPD. TA administration after exposure of C57BL/6 mice to CS reduced pulmonary edema, amount of inflammatory cytokines, and increased AMPK phosphorylation and Nrf2 expression [69]. Flavonoids, such as isoliquiritigenin (ILG) extracted from licorice roots, have also been reported to regulate Nrf2 and NF-κB pathways in COPD. Treatment of CS-induced COPD mouse model with ILG showed a decrease in inflammatory cytokine levels in BAL fluid and NF-κB in the lungs, as well as an increase in Nrf2 and HO-1, compared with the control group [70]. Treatment of normal human bronchial epithelial cells and BEAS-2B induced with CSE with alantolactone (ALT) extracted from *Inula helenium L*. reduced inflammatory cytokines, levels, and MDA (malondialdehyde), also known as an indication of oxidative stress, and activated Nrf2/HO-1 pathway [71].

### 3.3. Idiopathic Pulmonary Fibrosis (IPF)

IPF is a type of chronic interstitial disease characterized by a progressive decline in pulmonary function. It has a median survival of approximately 3–5 years after the diagnosis [72]. Currently, two antifibrotic drugs, pirfenidone, and nintedanib have been announced to slow the fibrotic process and decline in lung function in mild and moderate disease [73]. At the early stages of respiratory disease, inflammatory responses activate alveolar regeneration, resulting in fibrosis [74]. However, anti-inflammatory agents do not prolong the survival of patients with IPF. As alveolar epithelial cells become injured and die, the cells regenerate through differentiation or self-renewal program under normal conditions, whereas impaired regeneration activates transforming growth factor-β (TGF-β) signaling that is essential for fibrosis progression and interacts with the function of fibroblasts/myofibroblast [75]. Activation of fibrosis-associated signaling pathway promotes progression of epithelial-mesenchymal transition (EMT) and differentiation of fibroblasts into myofibroblasts that mediate the accumulation of extracellular matrix (ECM), including collagen [76].

In general, IPF has been known as one of the aging-related diseases, primarily occurring in patients aged over 60 years [77]. Aging not only causes a decrease in the functional capacity of the lungs but also profoundly influences tissue repair [78]. Since oxidative stress takes part in the development of cellular senescence-related aging, it can be the main risk factor for developing chronic disorders, such as inflammation and fibrosis [79]. Several studies indicated that IPF patients have a higher oxidant burden [80]. For example, in a histochemistry study, SODs were almost absent in fibrotic foci [81]; in meta-analysis evaluating the GSH level showed that patients with IPF have significantly lower GSH levels compared with controls [82]. TGF-β is one of the most well-known pro-fibrotic factors. It has been reported to increase the amount of ROSs and inhibit the antioxidant system, including oxidative stress or redox imbalance [83]. Macari et al. exhibited that oxidant/antioxidant imbalance in IPF fibroblasts was associated with downregulation of nuclear Nrf2 expression [84]. In animal research, bleomycin is one of the most frequently used agents to induce pulmonary fibrosis. Although bleomycin had been developed as an antineoplastic chemotherapeutic drug for many carcinomas and lymphomas, it has generated excessive ROSs through DNA strand breakage during its anticarcinogenic mode of action leading to pulmonary fibrosis in ~3–5% of patients [85]. Based on its side effect, including DNA breakage, excess ROS generation, and fibrosis, bleomycin is a suitable inducer for modeling pulmonary fibrosis in laboratory rodents. Ghatak and colleagues showed that bleomycin-induced TGF-β1/p-SMAD3 facilitates induction of NOX4 by its receptor, TGFRI, and impairs oxidant/antioxidant balance during fibrosis development. This pathway forms a feedback loop to upregulate ROS level, which induces cell invasion and deposition of pathological ECM components [86].

It has been indicated that bleomycin-induced pulmonary fibrosis showed suppression of Keap1-Nrf2 in vivo [87]. In another animal study, Nrf2-deficient mice showed a more severe and earlier lung fibrotic response to bleomycin than wild-type mice. The authors demonstrated that EMT-associated genes are significantly influenced by the loss of epithelial marker, E-cadherin, and the increase in myofibroblast markers, vimentin, and α-SMA, in Nrf2-deficient condition. In conclusion, this study suggested that Nrf2 protects against the EMT during fibrosis; therefore, Nrf2 may be a potential therapeutic target for IPF [88]. Furthermore, Nrf2 activators have been recently studied as a therapy for IPF [12]. For example, SFN influences IPF, reducing EMT. SFN is a phytochemical that is mainly found in cruciferous vegetables such as broccoli, cabbage, and Brussels sprouts and is known as an Nrf2 inducer [89]. In human IPF fibroblasts, SFN inhibited proliferation, migration, and expression of fibrosis-related proteins, whereas mRNA expression of NQO-1, EPHX, and HO-1 was increased. Although the authors of this study indicated that SFN has an antifibrotic effect through Nrf2 activity in human controls or IPF fibroblasts, they had not sought evidence that SFN exhibits antifibrotic effect in bleomycin-induced IPF mice [84]. Recently, however, some researchers have shown that SFN suppressed TGF-β-mediated EMT signaling in A549 cells. Additionally, SFN considerably attenuates pro-fibrotic proteins such as fibronectin and TGF-β, EMT markers, and early inflammation in vivo suggesting that it is an effective therapeutic agent [90,91].

Furthermore, rapamycin, an mTOR inhibitor, has frequently been investigated as a regulator of the Nrf2 signaling pathway in various cell types [92,93]. In paraquat-induced pulmonary fibrosis models, rapamycin attenuated fibrotic pathology in lungs after a decrease in expression of Col I, Col III, MMP2, and MMP9, which are well-known fibrosis-related genes [94]. In this research, rapamycin showed critical activation of Nrf2 and inhibition of Keap1 signaling, showing an antifibrotic effect. On the contrary, in another recent paper, the authors have shown that rapamycin does not block the accumulation of collagen in primary human lung fibroblasts compared with another mTOR inhibitor, AZD8055. They investigated the reason why rapamycin could not control collagen deposition and found that the mTOR/4E-BP1 axis is integral for collagen deposition, whereas upstream of mTOR, PI3K/Akt signaling, is unnecessary. Since 4E-BP1 are avidly phosphorylated and insensitive to rapamycin treatment, rapamycin had no effect on collagen synthesis [95]. These findings demonstrated that further studies on Nrf2 activator/regulator function in IPF are needed.

### 3.4. Asthma

Asthma is a chronic inflammatory disease characterized by reversible airflow obstruction, airway bronchial hyperresponsiveness to a variety of allergens or infectious organisms, leading to shortness of breath, cough, and wheezing [96]. The pathophysiology of asthma involves an inappropriate inflammatory response led by the T-helper type (Th)-2 lymphocytes to normally harmless airborne allergens [97]. Activated Th2 lymphocytes trigger IgE production in B cells, which release various cytokines, including IL-4, IL-13, and IL-5. Additionally, they mediate eosinophil activation and recruitment and mucus production [98]. Furthermore, oxidative stress has been reported to contribute to asthma development since ROS formation is a component of key mechanisms of asthma pathogenesis [99]. Inflammatory cells are the main source of ROSs in asthma. Antigenic challenges in asthmatic patients mediate the production of ROSs by eosinophils [100]. Under inflammation, stimulated eosinophils rapidly release extracellular DNA traps as an important mechanism of the innate immune response, trapping and killing pathogens [101]. Recently, it has been demonstrated that ROSs are involved in eosinophil extracellular traps in the asthma experimental model. Ovalbumin (OVA) has facilitated the establishment of allergic airway inflammation in vivo. Because oxidative stress is closely associated with allergic asthma, various studies suggested antioxidants as a therapeutic target for asthma [102,103,104]. Sussan et al. demonstrated that Nrf2 reduces the severity of allergic asthma in mice. As they activated Nrf2 with 2-trifluoromethyl-2′-methoxychalone (TMC), it significantly decreased the number of eosinophils and allergic-related cytokines such as IL-4 and IL-13 [102]. Recently, Pan and colleagues have shown that edaravone, a free radical scavenger, has a therapeutic effect in OVA-induced asthma mice [105]. Edaravone has strong antioxidative action: it attenuated ROS levels, recovered SOD, CAT, and GSH-Px, and activated the Keap1-Nrf2 pathway and HO-1 expression in the lungs [105]. Another Nrf2 activator, RTA-408, has been studied in OVA/ozone-induced acute asthma exacerbation. RTA-408 effectively reduced the number of inflammatory cells, including eosinophils and neutrophils. RTA-408 decreased pro-inflammatory cytokine production and reduced the percentage of IL-17+ γδT cells [106]. Although SFN had received significant attention as a possible intervention for airway inflammation, a proof-of-concept clinical study showed that SFN did not induce expression of Nrf2-regulated genes or have prospective effects with ozone exposure [107]. In addition, Sakurai et al. demonstrated that SFN activates histone deacetylase 2 (HDAC2), a key regulator of steroid response, via Nrf2 activation in CS-related asthma [108]. These studies suggest that Nrf2 activator is a potential drug candidate that has powerful anti-inflammatory and antioxidative effects in patients with asthma.

## 4. Conclusions

The Keap1-Nrf2 pathway is a major antioxidant defense mechanism that protects the lung against oxidative stress. Lungs are constantly exposed to various harmful substances, such as CS, dust, PM, and even viruses and bacteria, which may cause oxidative stress. Since oxidative stress has been closely related to human respiratory diseases, antioxidant systems, including Keap1-Nrf2, have been become an important therapeutic target for various respiratory diseases. The emerging role of the Keap1-Nrf2 regulation, indicating that the enhancement of defense mechanism attenuates inflammatory response, oxidative stress, fibrosis, and cellular death and ultimately has protective effects against the development of diseases in experimental models in Table 1. These trials to regulate the Keap1-Nrf2 pathway could potentially be developed into therapies for various respiratory diseases.

## Figures and Tables

**Table 1:** Keap1-Nrf2 regulation in respiratory diseases.

Respiratory Disease	Keap1-Nrf2 Regulator	Therapeutic Mechanism	Reference
ARDS	SCN-16	Inflammation reduction in LPS-induced ARDS model.	[44]
ARDS	Dasatinib	Inhibition of recruitment neutrophils, lymphocytes, and macrophages in LPS-administrated mouse model.	[46]
ARDS	Oridonin	Nrf2, HO-1, GCLM upregulation and NF-kB pathway inhibition in RAW 264.7.	[47]
ARDS	CDDO-Im	Nrf2, Gclc, NQO1, and GPX2 upregulation in hyperoxia-induced lung injury mouse model.	[42]
ARDS	Sulphoraphane	Reduction in hypoxic lung injury via enhanced energy metabolism in mitochondria and cardiovascular function.	[45]
COPD	Triterpene Acids	Reduction in pulmonary edema, inflammatory cytokines. Activation of AMPK phosphorylation and Nrf2 expression in CS-induced COPD mouse model.	[69]
COPD	Isoliquiritigenin	Reduced inflammatory cytokines, decreased NF-κB expression, increased Nrf2 and HO-1 expression in CS-induced COPD model.	[70]
COPD	Alantolactone	Inflammatory cytokine and MDA reduction, activation of Nrf2/HO-1 pathway in CSE-induced NHBE and Beas-2B.	[71]
IPF	Sulphoraphane	Regulation of TGF- β-mediated EMT signaling in A549 cells, and pro-fibrotic proteins (e.g., TGF-β1, vimentin), and inflammation in mice.	[74,75]
IPF	Rapamycin	Increase in Nrf2 signaling pathway in paraquat-induced IPF rat model.	[78]
Asthma	Trifluoromethyl-methoxychalone	Decrease in the number of eosinophils and allergic-related cytokines such as IL-4 and IL13 in OVA-induced asthma mice.	[86]
Asthma	Edaravone	Decrease in ROS levels, and restore antioxidants (e.g., SOD, CAT, and GSH-Px). Activation of Keap1-Nrf2 pathway and HO-1 expression in the OVA-induced asthma mice.	[89]
Asthma	RTA-408	Decrease in pro-inflammatory cytokine production and suppression of the percentage of IL-17+ γδT cells in OVA/ozone-induced acute exacerbation asthma model.	[90]
Asthma	Surphoraphane	Activation of Nrf2 and HDAC2 in cigarette smoking-related asthma mice.	[108]

## Data Availability

Not applicable.
